# A Comparison Study of the Tehran Norms to the Reference Norms on Children Performance of the Bayley III

**DOI:** 10.22037/ijcn.v16i1.29236

**Published:** 2022-03-14

**Authors:** Farin SOLEIMANI, Nadia AZARI, Adis KRASKIAN, Hossein KARIMI, Firoozeh SAJEDI, Roshanak VAMEGHI, Soheila SHAHSHAHANI, Nayere MEHDIPOUR SHAHRIVAR, Amin SHAHROKHI, Robab TEYMOURI, Masoud GHARIB, Mehdi NOROOZI

**Affiliations:** 1 Pediatric Neurorehabilitation Research Center, University of Social Welfare and Rehabilitation Sciences, Tehran, Iran.; 2 Biostatistician, Department of Counseling and Guidance, Azad Islamic University, Karaj Unit, Alborz, Iran.; 3 Dept of Speech Therapy, University of Social Welfare and Rehabilitation Sciences, Tehran, Iran.; 4 Faculty of Paramedicine, Mazandaran University of Medical Sciences, Sari, Iran.; 5 Substance Abuse and Dependence Research Center, University of Social Welfare and Rehabilitation Sciences, Tehran, Iran.

**Keywords:** Bayley Scales of Infant and Toddler Development, Development, Testing norms, Children

## Abstract

**Objectives:**

The Bayley Scales of Infant and Toddler Development (3rd ed.; Bayley III) are widely used to assess cognitive, language, and motor development of children aged 1–42 months. It is unclear whether or not the reference norms of the Bayley III are acceptable for use in other populations or lead to over- or underestimating the developmental status of target children. This study aimed to compare the Tehran norms to the reference norms.

**Materials & Methods:**

We used Bayley III norms to assess cognitive, language, and motor development of 1,674 healthy children from health care centers in Tehran. Differences between the scaled scores were calculated based on the Tehran and reference norms. A one-sample multivariate analysis of variance (MANOVA) was used to control the mean difference scores over all subtests. When MANOVA showed significant differences between the scaled scores based on the Tehran and reference norms, we used univariate analysis to see which subtest and age group led to these significant differences. Finally, the proportions of children with low scores (scaled scores <7 or -1 SD and <4 or -2 SD) based on 2 norms were compared using the McNemar test to determine the over- or underestimation of developmental delay.

**Results:**

The scaled scores based on the Tehran norms varied across values based on the reference norms in all subtests. The mean differences were significant in all 5 subtests (*p* < .05) with large effect sizes for receptive and expressive communication, fine and gross motor subtests of .20, .23, .14, and .25, respectively, as well as with a small effect size for the cognition subtest of .02. Large effect sizes for all age groups were found for cognition, expressive communication, and fine motor subtests. More children scored below 1 and 2 SD using the Tehran norms. Using the reference norms resulted in underestimation of developmental delay regarding cognitive, receptive and expressive communication, and fine and gross motor skills.

**Conclusion:**

Population-specific norms should be used to identify children with low scores for referral and intervention. The Tehran norms differed from the reference norms for all subtests, and these differences were clinically significant.

## Introduction

Globally, the potential for growth, cognitive, and socioemotional development is not being achieved in more than 200 million children under the age of 5 years (1). Early interventions are vital to prevent long-term sequels because the critical period of brain development occurs in the early years of life (2). Children in low socioeconomic status face poor health conditions, large family size, lack of home environmental stimulation, and fewer educational resources (3). The early experiences of the infant can affect its whole life since physical, social-emotional, and cognitive development in early childhood provides the basis for the child’s development in future years (4,5). Previous studies have determined that the developmental paths of children with different cultural backgrounds, even within the same country, are significantly different for motor and language skills (6). 

The Bayley Scales of Infant and Toddler Development (3rd ed.; Bayley III) are the most commonly used tool to assess early developmental status, specifically cognitive, language, and motor skills (7). The Bayley III is an objective test with stepwise guidelines, norm-referenced scores, and appropriate psychometric properties to measure and assess infants’ development in health care settings and for scientific research purposes; it was designed and normalized in the US. Given the factors affecting the development of infants, such as genetic features, child-rearing, social habits, ecological characteristics, socioeconomic factors, and the relationship between these factors (8), using reference norms in populations with different features and cultures appears to result in misclassification of developmental delay. Overestimating development leads to the non-referral of infants who need intervention and the loss of opportunity for early interventions, while underestimating development increases health care costs, parents’ concerns, and unnecessary referrals. 

Most studies conducted on child development in developing countries use tests adapted, designed, and validated for western countries (9-11). In some studies, “translating” is the only effort made (12, 13), which if not carried out in conjunction with the so-called “adaptation” process, it cannot alone be indicative of that region’s cultural traditions and may lead to misinterpretation of the results (14). Also, these adapted tests have limited value without knowledge of a normal variety for other populations. Adjustment studies have been conducted in many developing countries, mainly using translated and rarely adapted original tests (15-18). In terms of development, this could lead to misclassification when the cut-off points of developed countries are used to assess children in developing countries (14).

Many studies conducted on Bayley III have shown that the reference norms are inappropriate to use in other populations (19- 27). Most of these studies have focused on specific groups (such as preterm infants and 1 age group with small sample sizes), and specific population norms have not been used in these studies (19-26). Population norms were used in 17 age groups only in 1 study to determine the cut-off points of the Bayley III in Dutch children regarding cognitive, motor, and language domains compared to the reference norms (27). 

This study aimed to compare the Tehran norms of the Bayley III to the reference norms in cognitive, language and, motor scales of Persian-speaking children aged 1 to 42 months. 

## Materials &Methods

This cross-sectional study was conducted in Tehran. We used the Bayley III, in which the adaptation, psychometric properties, and the Tehran cut-off points were determined in previous studies (28, 29). According to the health care services, Tehran is divided into 3 geographical regions (i.e., north and east, center and south, and west), which collectively cover 60% of the health care visits and 98% of vaccinations for children aged newborn to 5 years. Sampling was in proportion to the population of children covered in each region. Inclusion criteria were apparently-healthy 1- to 42-month-old children and Persian-speaking. Normal development was defined as “any child born with no significant medical complications and is not currently diagnosed with or receiving treatment for cognitive, motor, language, or behavioral problems.” The exclusion criteria consisted of known developmental disorders, including attention deficit and hyperactivity disorder, autism spectrum disorders, chromosomal disorders, congenital anomalies, intellectual disability, receiving rehabilitation interventions, sensory disorders, such as hearing or visual problems. The examiners for this study were selected based on the following criteria: a degree in occupational therapy, speech therapy, or psychology, as well as at least 2 years of experience working with children and a desire to work with them. They then received training. **Data collection tools and methods:** After obtaining the assigned parents’ informed consent, mothers completed a demographic questionnaire, such as the date of birth, child health, and the parents’ education.

The Bayley III is an individually administered assessment tool that assesses developmental functioning in children aged 1 to 42 months in cognitive (91 items), receptive (49 items) and expressive (48 items) communication, and fine (66 items) and gross motor (72 items) domains (7). For practical purposes, this age range is divided into 17 age groups. Scaled scores are derived from the raw scores of the Bayley III. The range of the scaled scores is 1 to 19, with an SD of 3 and a mean value of 10. Therefore, a scaled score of 10 in each subtest indicates mean functioning in that age group and 7 one SD below the mean, and scaled scores of 4 represent the 2 SDs from the mean (7). The Persian version of the Bayley III was used in this study, which had its validity confirmed in a previous study (28). The forward-backward translation method was used to prepare the Persian version of the Bayley III, and, to increase its application in Iranian culture, modifications were made to the items, especially in the subtests of receptive and expressive communication for compatibility with grammar and language development in Persian-speaking children. Also, these modifications were made to the illustrations of the stimulus book as follows: changes in the receptive communication subtest: the games were replaced with the usual games played in Iranian culture. Given the words’ frequency in the period of language development in Persian-speaking children, the word “candy” was replaced with “cake” and “bird” with “fish,” and the illustrations were appropriately changed, and the tool “cup” was replaced with “handled glass.” Given that there is a vowel point to indicate possession in the Persian language, this form of pronouns was also added to the instructions, and the simpler and more popular form of the continuous tense, namely “to have + present tense,” was used. Changes were also made to the pronouns. Changes in the expressive communication subtest: Changes were made with respect to expressing continuous verb tenses, use of plural words, signs of possession in the Persian language, present tense verbs, and signs of future verbs. 

**Data analysis:** The differences between the scaled scores for all children in all subtests, based on the Tehran and reference norms, were calculated. A one-sample multivariate analysis of variance (MANOVA) was used to control the mean difference scores over all subtests. When MANOVA showed significant differences between the scaled scores based on the Tehran and reference norms, we used univariate analysis to see which subtest led to these significant differences. Because the mean differences might be age-dependent, the MANOVA (including all subtests) was separately performed for each age group in the next step. These results were evaluated and interpreted according to Cohen (30) effect size (η p2) (.06 or less is small, .07-.13 medium, and .14 or higher large).

Finally, the proportions of children with low scores (scaled scores <7 and <4), based on the Tehran and reference norms, were compared using the McNemar test. Analyses were performed using SPSS version 20 (SPSS Inc, Chicago, Ill, USA). 

The Ethics Committee of the University of Social Welfare and Rehabilitation Sciences approved this study (code: 801/93/18330/1).

## Results

In this study, 1,674 children were enrolled, of which 913 were boys (54.5%). The highest educational level of the mothers was at a moderate level (47%). Table 1 demonstrates background characteristics and 17 age group distribution of the participants. Table 2 shows the univariate MANOVA results with significant differences for all subtests and large effect sizes for receptive and expressive communication and fine and gross motor subtests of .20, .23, .14, and .25, respectively, as well as a small effect size for the cognition subtest of .02. 

The mean difference and partial eta squared values between the scaled scores based on the Tehran and reference norms are presented in Table 3. The smallest mean difference (equal to .01) was found for cognition for age group B (1 month 16 days-2 months 15 days), expressive communication for age group N (25 months 16 days-28 months 15 days), fine motor for age group Q (39 months 0 days-42 months 15 days), and gross motor for age group A (16 days-1 month 15 days) and D (3 months 16 days-4 months 15 days). For gross motor skills for age group Q (39 months 0 days-42 months 15 days), the largest mean difference of 2.77 was found, which is near to 1 SD based on the scaled scores.The effect sizes regarding the multivariate analyses are displayed in the second column in Table 3. For all age groups, large effect sizes were found for the differences between the scaled scores based on the Tehran and reference norms, but not consistently for particular subtests or definite age groups (Table 3). For cognition, expressive communication, and fine motor subtests, effect sizes were generally large for all age groups. For the receptive communication subtest, effect sizes were generally large, with the exception of 4 age groups. For the gross motor subtest, effect sizes were generally large, with the exception of 6 age groups. Using a scaled score of 7 (-1 SD) or 4 (-2 SD) as the cut-off point, McNemar tests showed that for all subtests, except for a scaled score of 4 for fine motor, significantly different rates of children with low scores were found using the Tehran and reference norms (Table 4). It means that fewer children scored below 1 or 2 SD in cognition, expressive, and receptive communication and fine and gross motor performance when using the reference norms instead of the Tehran norms. In addition, McNemar tests were performed on 4 age groups (Table 4). The proportions of children scoring below 1or 2 SD using the Tehran and reference norms varied significantly for all age groups. Therefore, using the reference norms, fewer children score below 1 and 2 SD than the Tehran norms.

## Discussion

In this study, a total of 1,674 children were assessed using the Bayley III. The present study, conducted on a large number of Persian-speaking children in Tehran, showed a significant difference between the Tehran and reference norms. Overall, the effect size of the difference between the Tehran and reference norms was large. There were significant rate differences in the analysis of developmental delay children. Using the reference norms leads to an overestimation of development in all subtests compared to the Tehran norms, as well as underdiagnosis of developmental delay. These findings showed important differences in children’s functioning and developmental levels between the 2 populations. Also, the results showed that over- or underestimation of development using these 2 norms was somewhat age-dependent, especially in 0-6 months groups. 

The same method used for the main reference norms was also used for standardization and normalization in Tehran, but our sample included only apparently-healthy children. Therefore, the difference may be due to the mixed sampling method in the reference norms. The inclusion of about 10% of high-risk and developmental disorders groups in the normative sample of the third edition in the US is the most likely cause for the overestimation, as this methodology tends to lower group means, increase SD, and, as a result, decrease the ability to detect developmental delay. On the other hand, the difference between the Tehran and reference norms may be due to differences in race, socioeconomic status (SES), parents’ ethnicity, and environmental factors. Previous studies have shown that even in a single country, the development of children with different ethnic backgrounds differs significantly with respect to acquiring motor (31) and language (6) skills. 

Another factor affecting children’s development is the parents’ education. Previous studies have shown that mothers’ education is related to the scores of children (32). In the US, the education level is measured based on the number of years of education; accordingly, 42% of the mothers have low levels of education, 30% moderate, and 28% high (7). In the present study, 38.5% of the mothers had low, 47% moderate, and 11.7% high levels of education. The difference in the pattern of the Tehran and reference norming sample regarding educational levels might also contribute to the differences between the norms.

Specific norms with cut-off point’s determination were done only in the Netherlands by Steenis et al. In this study, the scaled scores of the US and Dutch norms were compared in 1,912 children between the age groups of 16 days and 42 months and 15 days. The researchers found that using the reference norms led to overestimation of the gross motor development and underestimation of the cognitive, receptive, and expressive communication and fine motor development (27). Using the reference norms instead of the Tehran norms (such as Dutch norms), the study leads to misclassification of developmental delay, indicating the need for using norm-specific scores.

In another study conducted by Manandhar et al., using the Nepalese version of the Bayley III on 102 children aged 1-42 months, comparing the scaled scores of these children with the US (unlike our study) revealed that the mean scaled scores were lower than the mean scaled reference scores in cognitive, fine, gross motor domains (33). The results are not similar to our study, which may be due to the small sample size, rearing and cultural factors, parents’ educational level/SES, and ethnicity. Finally, the authors concluded that using population-specific norms instead of the reference norms used outside the US is recommended, even though creating such norms is time-consuming and expensive. 

Nguyen prepared the Vietnamese version of the Bayley III, tested it on 129 Vietnamese children, and determined its psychometric properties. The authors concluded that there were no significant differences in the performance of Vietnamese children against the US norms and argued that the US norms could be used for this version (34). This result is not similar to our study. The small sample size (7-9 children in each age group) and the simple comparison of the raw scores without any categorization based on the 2 versions might limit appropriate statistical comparisons in the study.

In other studies on the Bayley III, population-specific norms were not used, and the only mean scores of the special 2 groups were compared with the US norms. In a study conducted by Walker et al., 211 one-year-old Australian infants were randomly selected. The mean scores in all the subtests, except for the fine motor subtest, were significantly different from the reference norms. Therefore, the researchers suggested that specific cut-off points should be used for Australian children when administering the Bayley III (21). 

In a study conducted by Chinta et al. in Australia, 156 three-year-old term children were assessed using the Bayley scale and compared to the reference norms in terms of the mean scores in 5 domains. The mean scores were higher in the cognitive, fine motor, and receptive and expressive communication subscales, and there were no significant differences in the gross motor subscale. The highest difference was observed in the receptive communication subscale. Similar to our results, the researchers concluded that children with mild developmental delay might not be diagnosed when using reference norms and recommended the use of Australian norms in this population (20). Higher mean scores have also been reported in Australian children who had been assessed using the reference norms in a number of other IQ and developmental tests, showing that the results of these tests can be affected by cultural factors and the individual’s level of maturity and performance in the test (21, 35). The results also agree with preliminary studies conducted in different countries, which compared Bayley’s test results with the reference norms (19-23). 

In a study conducted by Krogh et al. in Denmark (2012), longitudinal data were collected at 4, 7, 10, and 13 months for 45 Danish infants; the results showed significant differences between the US and Danish scores in cognitive, language, and motor domains. The difference was particularly noticeable in receptive communication, such that Danish infants had a significant developmental delay in all the age groups compared to the US infants. According to the authors, 1 reason for this difference was the nature of Danish language phones, which make the process of learning this language more difficult, and the researchers suggested that care should be taken in using Bayley cut-off points in countries without normed scores specific to that society (23). In another study conducted by the same researchers (2016), 43 Danish children were tested using the Bayley III at 2 and 3 years. Despite the previous study, the results showed no significant differences between the scores of the Danish and American children in motor and cognitive domains, the Danish children scored higher in the language domain, perhaps because the Bayley III overestimated the language development of Danish children in this age band (36). Similar to our study, the mean scores of receptive and expressive communication were also higher than the reference norms, especially in the receptive communication subtest.

In 2014, Cromwell et al. assessed the validity of the Bayley III in Mali by administering the test in 167 healthy Malawian children, and, while extracting the normed scores, they used the standard Z-score to categorize developmental delay. According to their results, the mean scores of Malawian children aged less than 6 months were higher than those of their American counterparts in all the subtests, but after the age of 6 months, the US children showed higher mean scores. The researchers concluded that the reference norms were different in different populations and that the interpretation of the test in Malawian children based on the US norms led to errors in categorizing developmental problems, especially in cognitive and communication domains (24).

In a study in Taiwan, similar to our study, the results showed that the Bayley III overestimated the development of Taiwanese children, and the researchers recommended that the cut-off points should be raised to show a developmental delay in this population (22). 

Godamunne compared the cognitive and motor domains of the translated Bayley III in 150 Sri Lankan term children and found that at age 12 months, the Sri Lankan children scored significantly higher than the US children in the cognitive subtest––but lower in the gross motor subtest. In contrast, at 24 months old, the US children’s scores were higher than the Sri Lankan children on the cognitive scale, and there was no significant difference between them in their motor scores. Similar to our results, the score differences did not follow a fixed age-dependent pattern (26). 

According to these studies, using reference norms may not be appropriate in other populations since children from different countries develop at a lower, similar, or occasionally higher rate than the reference norms.

**Table 1:** Background characteristics of the sample (N=1674)

	N	Percent
**Gender**		
Boys	913	54.5
Girls	761	45.5
mother Educational level *		
Low	643	38.5
Moderate	788	47
High	243	14.5
**age groups**		
A: 16 days-1 month 15 days	53	3.2
B: 1 months 16 days-2 months 15 days	62	3.7
C: 2 months 16 days-3 months 15 days	63	3.8
D: 3 months 16 days-4 months 15 days	108	6.5
E: 4 months 16 days-5 months 15 days	58	3.5
F: 5 months 16 days-6 months 15 days	86	5.1
G: 6 months 16 days-8 months 30 days	159	9.5
H: 9 months 0 days-10 months 30 days	96	5.7
I: 11 months 0 days-13 months 15 days	138	8.2
J: 13 months 16 days-16 months 15 days	163	9.7
K: 16 months 16 days-19 months 15 days	116	6.9
L: 19 months 16 days-22 months 15 days	89	5.3
M: 22 months 16 days-25 months 15 days	80	4.8
N: 25 months 16 days-28 months 15 days	101	6.0
O: 28 months 16 days-32 months 30 days	84	5.0
P: 33 months 30 days-38 months 30 days	132	7.9
Q: 39 months 30 days-42 months 15 days	86	5.1

*‘Low educational level’ refers to special education, primary school, or pre-vocational secondary education (< 12 years); ‘medium educational level’ refers to senior general secondary education, pre-university education, or secondary vocational education (13–16 years); ‘high educational level refers to higher professional education or university (17+ years).

**Table 2:** MANOVA results per sub-tests over all age groups

	**Mean difference**	**P**	**95% CI**	**ηp2**
**Cognition**	-0/02	0/04	-0/07,0/03	0.02
**Receptive Communication**	-1/01	0/001	-1/16,1/00	0.20
**Expressive Communication**	-0/51	0/001	-0/57,0/46	0.23
**Fine Motor **	-0/29	0/001	-0/38,-0/20	0.14
**Gross Motor **	-0/73	0/001	-0/82,-0/66	0.25

Note. The Mean difference is calculated by the scaled score based on the Tehran norms minus the scaled score based on the US norms. Mean differences < 0 indicate that the score based on the US norms was higher than the scaled scores based on the Tehran norms. Mean differences >0 indicate that the scaled score based on the US sample is lower than the scaled score based on the Tehran sample. The number of degrees of freedom in all subtests is 1.

**Table 3:** Mean differences (SD), and Partial eta squared values for all subtests per Bayley-III age groups

**Age** **group**	**Cognition**		**Receptive Communication**		**Expressive Communication**		**Fine Motor**		**Gross Motor**	
	**Mean difference** **(SD)**	**η p2**	**Mean difference** **(SD)**	**η p2**	**Mean difference** **(SD)**	**η p2**	**Mean difference** **(SD)**	**η p2**	**Mean difference** **(SD)**	**η p2**
**A**	-1.47(0.3)	**0.55**	-0.58 (0.2)	**0.35**	0.03(0.01)	0.15	0.58(0.21)	**0.15**	0.01(.001)	0.10
**B**	0.01(0.01)	0.65	-0.54 0.21)	**0.45**	1.61(0.87)	**0.25**	0.83( 0.15)	**0.30**	0.17(0.03)	0.21
**C**	-0.50(0.10)	**0.72**	-0.26(0.11)	**0.52**	0.68(0.14)	**0.52**	0.52(0.12)	**0.40**	0.58(0.16)	**0.28**
**D**	-0.96(0.07)	**0.44**	0.75(0.08)	**0.14**	0.15(0.12)	0.49	-0.35(0.11)	**0,03**	-0.01(0.12)	0.12
**E**	-0.44(0.11)	**0.23**	0.96(0.12)	**0.20**	0.41(0.19)	**0,16**	-0.25(0.11)	**0,33**	-1.68(0.14)	**0.30**
**F**	-0.25(0.08)	**0.25**	2.22(0.08)	**0.21**	-0.31(0.06)	**0.20**	0.73(0.05)	**0.18**	-0.62(0.06)	**0.21**
**G**	0.55(0.08)	**0.11**	1.42(0.06)	**0.19**	0.07(0.04)	0.44	0.54(0.04)	**0.28**	-0.81(0.03)	**0.22**
**H**	-0.34(0.09)	**0.60**	1.13(0.08)	**0.50**	-0.05(0.07)	0.35	-0.05(0.07)	0.44	-1.15(0.04)	**0.21**
**I**	0.89(0.05)	**0.32**	1.44(0.05)	**0.12**	0.40(0.04)	**0.14**	0.08(0.07)	0.18	-0.05(0.10)	0.10
**J**	1.04(0.06)	**0.44**	1.51(0.04)	**0.24**	0.74(0.07)	**0.38**	1.49(0.09)	**0.33**	2.06(0.07)	**0.07**
**K**	0.23(0.10)	**0.17**	0.83(0.07)	**0.22**	-0.43(0.08)	**0.55**	0.70(0.11)	**0.43**	1.12(0.10)	**0.30**
**L**	0.16(0.11)	0.32	0.95(0.12)	**0.02**	0.07(0.09)	0.44	0.58(0.09)	**0.10**	0.80(0.09)	**0.19**
**M**	-0.36(0.12)	**0.23**	1.31(0.10)	**0.13**	0.33(0.11)	**0.13**	0.45(0.12)	**0.21**	1.08(0.10)	**0.16**
**N**	-0.05(0.12)	0.21	0.89(0.07)	**0.27**	-0.01(0.06)	0.44	0.49(0.06)	**0.33**	1.70(0.13)	**0.20**
**O**	-0.25(0.13)	0.04	1.13(0.12)	**0.14**	1.92(0.09)	**0.14**	1.10(0.13)	**0.15**	2.60(0.12)	**0.06**
**P**	-0.19(0.11)	0.21	1.33(0.07)	**0.09**	1.65(0.12)	**0.27**	-2.16(0.33)	**0.11**	2.52(0.09)	**0.22**
**Q**	-0.09(0.13)	0.19	2.05(0.12)	**0.10**	1.70(0.17)	**0.12**	0.01(0.32)	0.16	2.77(0.10)	**0.11**

Note. The Mean difference is calculated by the scaled score based on the Tehran norms minus the scaled score based on the US norms. Mean differences < 0 indicate that the score based on the US norms was > the scaled scores based on the Tehran norms. Mean differences >0 indicate that the scaled score based on the US sample is < the scaled score based on the Tehran sample. Effect sizes are all statistically significant, p < .01, except those not bold.

**Table 4:** Proportion of children with low scores based on the US or the Tehran norms

	**Us norms** **<- 2SD %**	Tehran **norms** **< -2SD %**	**Us norms** **< -1SD %**	Tehran **norms** **< -1SD %**
**All age groups**				
Cognition	1.7	3.1*	12.8	19*
Receptive Communication	0.5	3.4*	7.2	19.6*
Expressive communication	1.2	3.3*	12.7	21*
Fine Motor	1.2	2.9	8.2	17.8*
Gross Motor	1.8	3.4*	12.1	19.2*
**4 Age groups**				
**0–6 months 15 days**				
Cognition	1.1	1.9*	30.6	23.1*
Receptive communication	1	1.9*	9.3	19.4*
Expressive communication	0.9	3.7*	11.1	24.1*
Fine Motor	0.9	0	7.4	12*
Gross Motor	1.1	0	3.7	20.4*
**6 months 16 day -13 months 15 days**				
Cognition	2.9	4.3*	9.4	15.2*
Receptive Communication	1.4	5.8*	7.2	15.9*
Expressive communication	2.2	5.1*	15.2	21*
Fine Motor	2.2	4.3*	16.7	18.1*
Gross Motor	1.9	5.8*	22.5	15.2*
**13 months 16 day -25 months 15 days**				
Cognition	0.6	2.5*	5.5	19.6*
Receptive Communication	1.2	3.7*	9.8	20.9*
Expressive communication	1	1.8*	3.7	23.3*
Fine Motor	1	2.5*	3.7	20.2*
Gross Motor	1.6	3.7*	8	17.8*
**25 months 16 day -42 months 15 days**				
Cognition	0	1.2*	4.8	19*
Receptive Communication	0	3.6*	3.5	13.1*
Expressive communication	0	4.8*	3.6	11.9*
Fine Motor	0	3.6*	7.1	19*
Gross Motor	0	3.6*	1.2	17.9*

Note. Scaled scores of < -1SD correspond to scaled scores <7 indicating a low score which may reflect a mild to moderate developmental delay in the subtest domain and scaled scores of <- 2SD correspond to scaled scores <4, which may indicate a moderate to severe delay in the domain examined.* p < .01

## Limitation

The findings of other studies on the Bayley III revealed that some adaptations are needed to make the Bayley III appropriate for other countries. Thus, in the Persian version, besides translation, some changes were made to all subtests, especially to the language scale in accordance with Iranian culture and Persian language development (28). However, we did not assess whether the original sequence of items of the Bayley III, in which the items increase in difficulty, would be appropriate for the assessment of Iranian children and whether the same item arrangement could be applied. Thus, we suggest that the level of difficulty for adapted items be determined in accordance with the item’s increasing difficulty pattern. Another suggestion is to provide a language scale for other Iranian languages such as Turkish, Kurdish, and Baluch.

## In Conclusion

Although the Bayley III is known as an accurate developmental assessment tool, standardized norms are required in each population to enable clinical and research application. Among Persian-speaking children in Tehran, the Tehran norms should be used. Using the reference norms of the Bayley III in Tehran children led to an underestimation of developmental delay. 

## Author’s Contribution

Soleimani F, Azari N, Kraskian A, and Norouzi M had substantial contributions to the conception and design of the work; they had substantial contributions to acquisition, analysis, interpretation of data and drafting the work and revising it critically for important intellectual content; they had also contributed for final approval of the version to be published; and they have agreement to be accountable for all aspects of the work in ensuring that questions related to the accuracy or integrity of any part of the work are appropriately investigated and resolved. Vameghi R, Sajedi F, Shahshahani S, Karimi H, Shahrokhi A, Teymouri R, Gharib M, and Mehdipour N had substantial contributions to the conception, design, drafting and revising the work; they had contributed for final approval of the version to be published; and they have agreement to be accountable for all aspects of the work in ensuring that questions related to the accuracy or integrity of any part of the work are appropriately investigated and resolved.
